# Antitumor Potential of Immunomodulatory Natural Products

**DOI:** 10.3390/md20060386

**Published:** 2022-06-08

**Authors:** Genoveffa Nuzzo, Giuseppina Senese, Carmela Gallo, Federica Albiani, Lucia Romano, Giuliana d’Ippolito, Emiliano Manzo, Angelo Fontana

**Affiliations:** 1 Bio-Organic Chemistry Unit, Institute of Biomolecular Chemistry-CNR, Via Campi Flegrei 34, 80078 Pozzuoli, Italy; giusi.senese@icb.cnr.it (G.S.); carmen.gallo@icb.cnr.it (C.G.); f.albiani@icb.cnr.it (F.A.); l.romano@icb.cnr.it (L.R.); gdippolito@icb.cnr.it (G.d.); a.fontana@icb.cnr.it (A.F.); 2 Department of Biology, University of Naples Federico II, Via Cinthia–Bld. 7, 80126 Napoli, Italy

**Keywords:** natural products, marine products, immunomodulators, antitumor, anticancer

## Abstract

Cancer is one of the leading causes of death globally. Anticancer drugs aim to block tumor growth by killing cancerous cells in order to prevent tumor progression and metastasis. Efficient anticancer drugs should also minimize general toxicity towards organs and healthy cells. Tumor growth can also be successfully restrained by targeting and modulating immune response. Cancer immunotherapy is assuming a growing relevance in the fight against cancer and has recently aroused much interest for its wider safety and the capability to complement conventional chemotherapeutic approaches. Natural products are a traditional source of molecules with relevant potential in the pharmacological field. The huge structural diversity of metabolites with low molecular weight (small molecules) from terrestrial and marine organisms has provided lead compounds for the discovery of many modern anticancer drugs. Many natural products combine chemo-protective and immunomodulant activity, thus offering the potential to be used alone or in association with conventional cancer therapy. In this review, we report the natural products known to possess antitumor properties by interaction with immune system, as well as discuss the possible immunomodulatory mechanisms of these molecules.

## 1. Introduction

Natural products (NPs) have historically been the active constituents of traditional medicines and represent a major source of modern therapeutic agents [1]. According to the World Health Organization (WHO), 75% of humanity relies on natural remedies for health care [2].

Currently, significant revival of interest in NPs as a source for novel drugs is occurring for their matchless structural variety and for the usual small molecular weight that make these molecules particularly suitable for pharmacological development [3]. According to Newman and Cragg, from 1981 to 9/2019, almost 33% of the new approved drugs were NP or natural derivate (ND), and over 22% were nature-inspired synthetic compounds [4]. In cancer research, 40 of the 75 small molecules (53.3%) identified from 1946 to 1980 are NP or ND [4].

Tumor is one of the primary cause of death. It is responsible for approximately 7.6 million deaths in the world every year, with the dramatic prediction of 13.1 million by 2030. NPs have inspired or provided a large fraction of anticancer drugs. Today, more than 60% of anticancer drugs in clinical use originate from NPs derived from plants, marine organisms, and microorganisms [5]. Furthermore, NPs are often molecules that are inaccessible by alternative ways, such as paclitaxel (Taxol), a complex diterpenoid compound that was first isolated from *Taxus brevifolia* [6] and approved by the Food and Drug Administration (FDA) in 1992. Another example is Trabectedin (ET-743) (Yondelis^®^, approved in 2015), one of the few marine natural products commercially available, whose tetrahydroisoquinoline core is prepared by semi-synthesis from cyanosafracin B produced by cultures of the bacterium *Pseudomonas fluorescens* [7,8].

**Figure 1:**
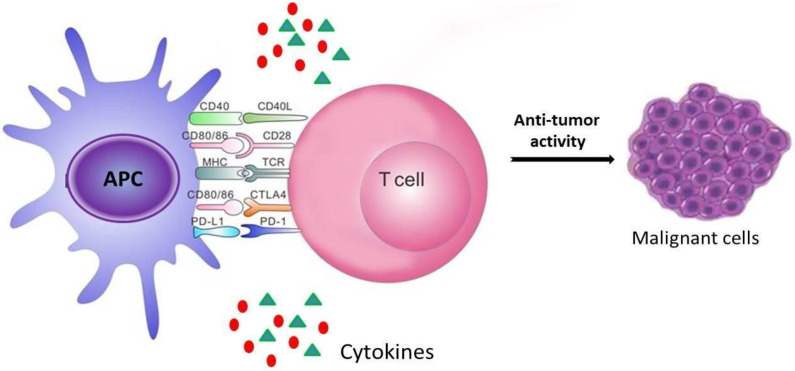
The “immune synapse”: APC: antigen presenting-cell; PD-L1: programmed death ligand-1; PD-1: programmed death-1; CTLA-4: cytotoxic T-lymphocyte antigen-4; TCR: T cell receptor; MHC: major histocompatibily complex; CD28, CD40, CD40L, CD80/86: cluster of differentiation.

Conventional anticancer chemotherapy has been historically supposed to act through direct killing of tumor cells. Cytotoxic drugs interfere with basic cell functions leading to tumor cell death [9]. However, most of the anticancer drugs showed toxic effect to both cancer and healthy cells. In recent years, the importance of the immune system to combat tumor has been discovered and the interest on the exploitation of natural immunomodulatory substances in combination with usual cancer treatments increases, with the aim to ameliorate the immunological reaction against tumors and decrease the chemotherapy suppressive impact. The defense against cancerous cells comprises a dynamic and orchestrated interplay of innate and acquired immune response. Immunoediting is the phenomenon that regulates the evolution of tumors by the immune system. This dynamic process consists of three phases and starts with immunosurveillance followed by tumor progression and escape. In the first stage, a crucial inflammatory response generated by the immune system is necessary to recognize and eliminate the early originated cancer cells [10]. The main immune cells recruited for tumor defense are the antigen presenting cells (APC), such as dendritic cells (DCs) that play a pivotal role in detecting tumor cells and coordinating tumor eradication (Figure 1). DCs display antigen complexed with major histocompatibility complexes (MHC) and co-stimulatory molecules on their surfaces that allow the interaction with T cells. The release of chemical messengers or inflammatory cytokines such as interferon γ (IFN-γ) and interleukin 12 (IL-12) prime T helper 1 (Th1) response until triggering of cytotoxic T lymphocytes (CTLs). Nevertheless, cancer cells can escape immune control by several strategies shifting into equilibrium condition between cancer expansion and apoptosis. Among the immunotherapy plans, immune checkpoint therapy has taken on great relevance. The PD-1/PD-L1 checkpoint represents a crucial immunosuppressive arbiter in tumor immune microenvironment (TME) and is mainly related with IFN-γ signaling. Proinflammatory cytokines as INF-γ induce PD-L1 expression, an essential process to control the immunological functions. The physiological role of this dynamic process is to prevent an immune response from being too dangerous for healthy cells. Checkpoint inhibitors targeting PD-1/PD-L1 axes are antibodies blocking the “off” signal on T cells, thus favoring T cell activation against tumors. The better results in clinical practice were observed with checkpoint proteins block in melanoma and non-small-cell lung cancer (NSCLC) [11]. Nevertheless, only a fraction of the patients responds to these therapies and there are off-target side effects due to the treatment [12,13].

Although the field of immunotherapy is still growing, the discovery of immunomodulatory NPs able to stimulate the innate immunity cells represent the new frontier for tumor therapy. Zhong et al. have recently reviewed natural products from herbal medicines with immunomodulatory potential as immune checkpoints inhibitors [14]. Other studies have demonstrated that also some conventional chemotherapeutic agents can also affect the immune system by different mechanisms. Some cytotoxic drugs determined immunogenic cell death of cancer cells, resulting in the upregulation of damage-associated molecular patterns (DAMPS) triggering phagocytic activity of debris and promoting DC maturation and initiation of antitumor reaction [9]. The same Paclitaxel has been reported to induce DC activation (through TLR4 and MyD88) in mice [15,16] and recently different proofs indicated that antitumor drugs, as anthracyclines, induced an immunogenic apoptosis favoring the engulfment of apoptotic debris by DCs with cytotoxic CD8+ T cells activation [17]. For these reasons, alternative therapies to cytotoxicity that use the immune system to defeat and eliminate tumor cells are spreading.

Many authors have previously reviewed naturally occurring immunomodulators with antitumor activity [18,19,20,21,22,23]. Immunomodulatory potential and mechanism of action of natural compounds have also been discussed for metabolites from plants and microorganisms specifically [17,18]. However, a comprehensive report is still missing. In this review, we focused on NPs with immunomodulatory and antitumor properties from several sources. Special attention is paid to the discovery and the mechanism of action of immunomodulatory anticancer compounds from marine organisms.

## 2. Terrestrial Environment

Table 1 collects immunomodulatory natural products with antitumor properties derived from terrestrial source herein reported.

### 2.1. Plant Compounds

In plants, antitumor and immunomodulating substances are abundant. Numerous phytochemicals displayed promising activity with few side effects in the therapy of several tumors [24].

#### 2.1.1. Terpenes

Many studies reported the potential features and utilization of extracts of terrestrial plants for the development of drugs for several diseases including tumors. 

**Figure 2:**
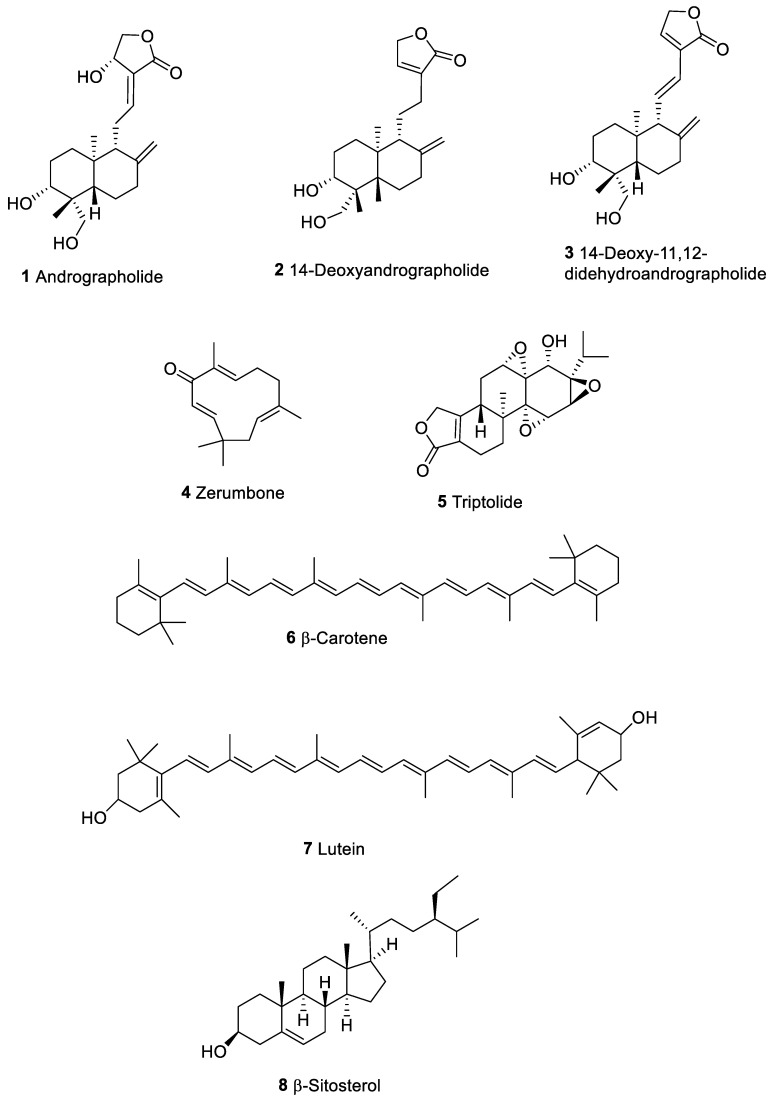
Terpenes from plants with anticancer and immunomodulatory properties.

*Andrographis paniculata* (Acanthaceae), known as “kalmegh”, is a medical plant of Ayurveda (Indian subcontinent) that is extensively cultivated in Asia. From leaves and stems of this plant, active phytochemicals, useful as anti-inflammatory [25], antiviral [26], anticancer [27], and immunostimulatory [28], were extracted. The organic extracts of *A. paniculata* and a few isolated metabolites displayed growth inhibition and differentiation activity on murine leukemia cells [27]. Andrographolide (**1**, Figure 2), the most abundant diterpenoid of the *A. paniculata* showed cytotoxic activity against KB (human epidermoid carcinoma) and P388 (lymphocytic leukemia cells) [28]. Andrographolides also stimulated antigen specific and non-specific immune response in mice [28]. Kumar et al. [29] confirmed the immunomodulatory activity of *A. paniculata* extracts and their constituents in human immune cells and antitumor activity in human tumor cells. However, between the diterpene compounds that have been isolated from the extract, andrographolide (**1**), 14-deoxyandrographolide (**2**), and 14-deoxy-11,12-didehydroandrographolide (**3**), only **1** significantly inhibited the proliferation of cancer cells with GI_50_ (concentration required to inhibit the 50% growth) values between 10 and 28 µM [29].

Among active terpenes, zerumbone (**4**, Figure 2), a sesquiterpenoidic derivative found in the rhizomes of *Zingiberaceae* plants, showed different pharmacological properties like antioxidant, antibacterial, antipyretic, anti-inflammatory, immunomodulatory, as well as anti-neoplastic [30,31]. This compound exerted its antitumor activity significantly suppressing expansion and metastasis by modulating nuclear factor kappa of activated B cells (NF-kB), protein kinase B (PKB), interleukin 6/janus kinase 2/signal transducer, activator of transcription 3 (IL-6/JAK2/STAT3), and their downstream target proteins. The immunomodulatory activities of zerumbone is suggested to be due to the α, β-unsaturated carbonyl-based part of the molecule [32]. Various in vivo and in vitro experiments proved **4** as a powerful antitumor metabolite [33], showing its impact on the mechanisms of tumor signaling. Studies to investigate the immunomodulatory properties of zerumbone have been mainly focused on mitogen-activated protein kinase (MAPK) and NF-kB pathways, NO production, and inflammation [34]. Zerumbone determined the suppression of different pro-inflammatory mediators as nitric oxide (NO), cyclooxygenase-2 (COX2), prostaglandin E2 (PGE2), and inducible nitric oxide synthase (iNOS) in RAW264.7 macrophages [35]. According to Eguchi et al. [36], this molecule highlighted immunosuppressive activity inhibiting activator protein 1 (AP-1) and NF-kB in the THP-1 cellular line. The effect of Zerumbone on human promyelocytic leukemia cells (HL-60) was reported to be due to the inhibition of 12-*O*-tetradecanoylphorbol-13-acetate (TPA)-induced super oxide anion generation from NADPH oxidase. Enhanced arrest of G2/M cell cycle was evident with a decreased cyclin B1/CDK1 ratio [37]. This compound was also evaluated on human tumor cells of ovary (Coav-3), breast (MCF-7), and HeLa cells (IC_50_ value of 20.30, 24.30, and 27.7 μM respectively) in addition to hamster ovaria normal cells [38]. Enhanced level of CASP3 was evident in zerumbone-treated HeLa, associated to cellular morphological features of apoptosis [38]. In addition, Yodkeeree et al. proved that increase of TNF-related apoptosis-inducing ligand (TRAIL) caused apoptosis of HCT116 cells (colon adenocarcinoma) [39]. To date, several pre-clinical trials seem to support zerumbone as promising immunomodulatory and preventive chemotherapeutic molecule. However, clinical studies on this compound have not been reported and further investigations are necessary to establish the therapeutic potential of zerumbone.

The diterpenoid triptolide (**5**, Figure 2) [40], isolated from Chinese Tripterygium Hook F (TWHF), is reported to inhibit proliferation of mice and human T lymphocytes [41,42] via the block of interleukin 2 (IL-2) production [42]. This compound inhibited several inflammatory mediators such as interleukin 1 (IL-1), tumor necrosis factor α (TNFα)*,* IL-6, interleukin 8 (IL-8), and PGE2. Downregulation of IL-8 expression was connected to the nuclear block of transcriptional activation of NF-KB [43]. Triptolide induces apoptosis of cancer cells and sensitize solid tumor cells toward TNFα-dependent apoptosis, by inhibition of NF-kB [44]. NF-kB is a protein transcription factor that binds DNA, regulates the expression of many biologically important genes such as those encoding inflammatory cytokines, and plays a role in the control of apoptosis. It has been described that triptolide inhibited transcriptional activation of NF-kB but not the DNA binding of NF-kB [42]. At concentrations between 5 and 20 ng/mL, this compound reduced cell viability by 40–70% in several solid tumor cell lines [44]. Triptolide can also induce modifications of the cell cycle checkpoints that enhances apoptosis in cancer cells by blocking p21-mediated growth arrest [45]. Amino acid derivatives of triptolide have been patented as immune modulators and antitumor compounds [46]. Phase II clinical studies of a triptolide analog in patients with refractory pancreatic cancer are available (A Phase II, International Open Label Trial of Minnelide™ in Patients With Refractory Pancreatic Cancer (MinPAC). Available online: https://clinicaltrials.gov/ct2/show/NCT03117920?term=triptolide&cond=cancer&draw=2&rank=2 (accessed on 13 April 2022)).

Carotenoids, tetraterpenoid organic pigments typically found in plants or other photosynthetic organisms such as algae and some species of bacteria, are characterized by different immunomodulatory activities. β-Carotene (**6**, Figure 2) is the main carotenoid isolated from dietary plants. This substance displayed immune-activating properties by triggering of natural killer cells (NK), increasing the number of leukocytes, CD4/CD8 ratio and upregulating MHC I proteins [47]. The anticancer property of lutein (**7**, Figure 2), characterized by two hydroxyl groups in the beta carotene end rings, was assessed in murine mammary tumor model. The molecule showed stimulatory impact on IFN-γ mRNA expression, downregulating interleukin 10 (IL-10) in splenocytes [48,49] along with apoptosis induction. According to World Cancer Research Fund/American Institute for Cancer Research (WCRF/AICR), foods containing carotenoids are considered protective against lung tumors [50,51]. The co-administration of carotenoids with traditional cytotoxic agents synergistically enhanced their cytotoxicity in Caco-2 cells, probably by inhibiting the function of the ATP-binding cassette (ABC) transporters. Although carotenoids showed a low cytotoxicity in cells with Multi Drug Resistance (MDR) with IC_50_ values between 100 and 200 μM, they can increase the cytotoxicity of chemotherapeutic drugs in human MDR1 expressing cells [52]. This observation revealed that carotenoids can be used in combination with anticancer drugs to enhance the efficacy of traditional drugs and reverse multidrug resistance (MDR) in cancer cells.

Other phytochemicals with a remarkable anticancer activity are phytosterols. β-sitosterol (**8**, Figure 2) favored apoptosis of colon tumor HT-29 and prostate cancer LNCaP cells at concentration of 16 µM and these effects were concomitant with reduction of cellular sphingomyelins and increase of ceramides amounts [53]. Mixtures of sterols and sterolins enhanced the cellular responsiveness of T lymphocytes both in vitro and in vivo experiments [54]. Dietary phytosterols have been recognized as immunomodulatory and chemopreventive compounds. Bouic [54] described the role of phytosterols (mixture of β-sitosterol **8** and its glucoside derivative, Figure 2) on human lymphocyte, along with production of a cytokine profile suggestive of a selective effect on Th1 (IL-2 and Tumor Necrosis Factor γ). Further investigation on β-sitosterol glucosides highlighted enhanced NK cell-dependent cytotoxicity against transformed cell lines [54]. Phytosterols also impact on macrophage activities; β-sitosterol has been reported to reduce NO release by phorbol ester from RAW264.7 macrophages, potentially correlated with the impairment of iNOS levels and NF-kB activation [55]. β-sitosterol also suppressed P388D1/MAB macrophages growth and inflammatory PGE2 secretion [56].

#### 2.1.2. Phenolic Compounds

Natural phenolic acids are well known for the antioxidant, anti-inflammatory and antimutagenic activities, as well as apoptosis inducted cytotoxic properties, and a suppressor effect on cancer attack and angiogenesis [45]. In addition to their phenolic antioxidants (PhOH) capability of transferring electrons and protect against pathogens, flavonoids represent a relevant class playing a crucial role in the regulation of diverse cell activities including production of chemokines for attraction of immune effectors into the tumor environment [15].

**Figure 3:**
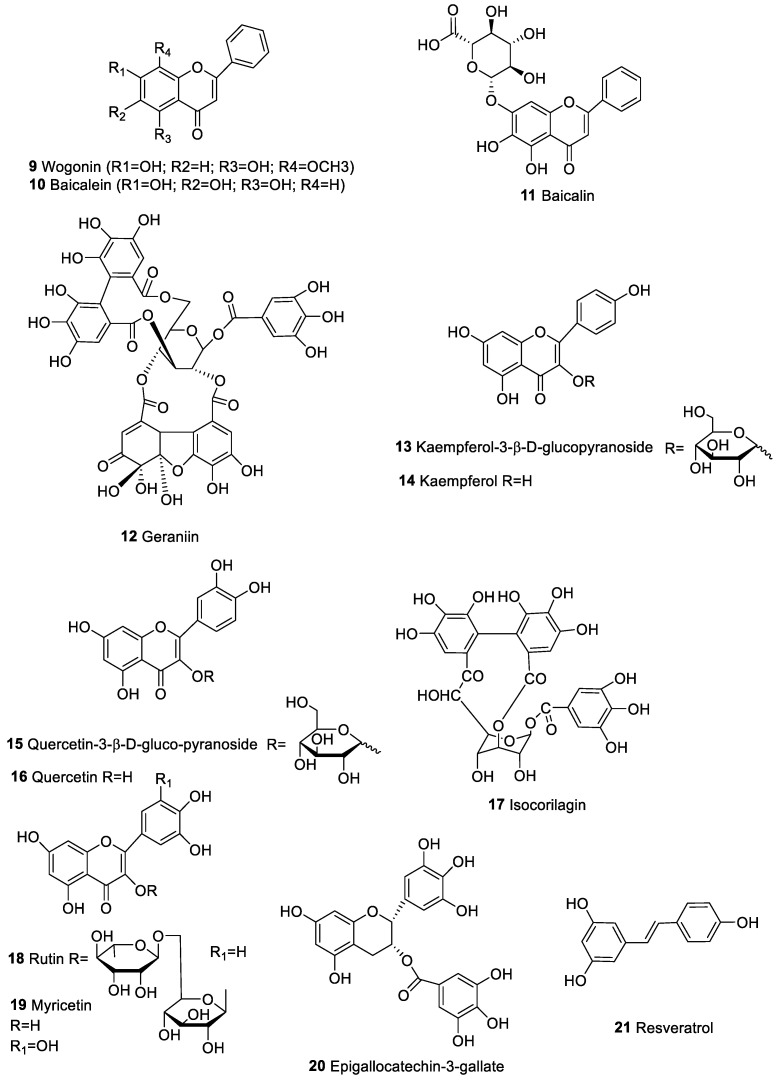
Phenolic compounds from plant with anticancer and immunomodulatory properties.

Many studies reported that the extract of the plant *Scutellaria baicalensis* controls cancer proliferation by cell cycle regulation. The low toxic effect toward non-malignant cells made *S. baicalensis* appealing as a source of novel antitumor drugs. *S. baicalensis* showed a cytostatic activity on different tumor cell lines in vitro [57] and in vivo in mouse cancer models [58]. The major constituents of *S. baicalensis* radix are flavonoid derivatives as baicalin, baicalein, wogonin, and wogonin-7-0-glucoronide (Figure 3). Among these flavones, wogonin (**9**), baicalein (**10**), and baicalin (**11**) inhibited the proliferation in many human tumor cell lines between 20 and 200 μM, based on the tumor cells evaluated. A detailed summary of these studies is discussed by Min Li-Weber [59]. Significant decrease in cyclin D1 protein level (regulatory protein of the cell cycle) in wogonin-treated breast tumor cell lines and in baicalein-treated prostate tumor cell lines has been also reported [60]. However, only for baicalein-treated lung cancer cell lines, a concomitant reduction of cyclin D1 and B1 levels was noticed [60]. In flavonoids-treated leukemia cell lines, the cell cycle was reduced at G2/M state [61]. In the case of wogonin or baicalein, the percentage of cells in G1 phase increased while in S phase decreased [62,63]. Lamer-Zarawska et al. [60] highlighted that the antitumor effect of flavones found in root of the *S. baicalensis* (wogonin, baicalein, and baicalin) was related to reduction of high-level reactive oxygen species (ROS), reduction of inflammatory reactions, and NF-kB activation. Flavonoids as wogonin, baicalein, and baicalin have been shown also to protect tissues from chronic inflammation associated to cancer, both in in vitro and in vivo models [59,64,65,66]. The anti-inflammatory effect of the *S. baicalensis* flavones is due to the inhibition of cytokines [67] and NO production via down-regulation of several inflammation-associated genes [64], such as iNOS [68], cyclooxygenase (COX) [68,69], and lipooxigenase (LOX) [68]. NO, a highly reactive free radical and its synthase NOS are ubiquitous in malignant tumors and are known to exert both pro- and antitumor effects [64,68,70,71]. Recently, Mengyun Ke et al. [72] showed that baicalein and baicalin flavonoids stimulated the T cell-mediated immune response against tumors through reduction of PD-L1 expression in hepatocellular carcinoma (HCC) and these effects were mediated by STAT3 activity inhibition. Therefore, baicalein and baicalin decreased STAT3 activity, downregulated IFN-γ-induced PD-L1 expression, and subsequently restored T cell sensitivity to kill tumor cells. These findings provide novel insight into the anticancer effects of baicalein and baicalin through which tumor growth was inhibited by PD-L1 downregulation, suggesting that these flavonoids have great potential for clinical treatment.

Fruit of Emblica (*Phyllanthus emblica Linnaeus*), a euphorbiaceous plant broadly present in subtropical and tropical zones of China, India, Indonesia, and the Malay Peninsula, exhibited antioxidant [73], hypolipidemic [73], and hypoglycemic activities [74], and antimicrobial [75] and anti-inflammatory properties. Recently, in vitro analysis also showed that this fruit was able to mitigate the immunosuppressant activity of chromium in rat lymphocytes [76]. Emblica extracts presented peculiar cytotoxicity to different tumor cell lines in vitro and in vivo [77,78]. Liu et al. [79] investigated immunomodulatory and antitumor activity of six phenolic molecules—geraniin (**12**), kaempferol 3-β-d-glucopyranoside (**13**), quercetin 3-β-d-gluco-pyranoside (**15**), isocorilagin (**17**), quercetin (**16**), and kaempferol (**14**)—found in Emblica fruit on mice splenocytes, human breast tumor lines (MCF-7), and human embryonic lung fibroblast (HELF) [79]. Significant stimulatory activities were detected for geraniin (**12**) and isocorilagin (**17**), which displayed major cytotoxicity toward MCF-7. Moreover, it was reported that isocorilagin showed potent cytotoxicity toward HELF, whereas geraniin, quercetin, kaempferol, and the related glycosides displayed minor effect to HELF cells [79].

Persimmon (*Diospyros kaki* L.) leaves, an Asian traditional medicine agent, containing mainly flavonoids, have been also described for their beneficial pharmacological effects including antitumor [80], hypoglycemic [81], antioxidant [82], and anti-inflammatory [82]. Li Chen et al. [83] identified four main flavone and flavonol glycosides, rutin (**18**), quercetin (**16**), kaempferol (**14**), and myricetin (**19**), described to have anticancer effect to leukemia, colorectal cancer, neuroblastoma, melanotic melanoma [84], and human prostate cancer cell line [85]. In this study, they found that persimmon leaves promoted serum interleukin 18 (IL-18) amount and NK cell cytotoxicity, suggesting activation of macrophages, and also favoring the NK cell-mediated antitumor response [83].

Analogously, tea plant *Camellia sinensis* exhibited multiple health benefits [86,87,88] and epidemiologic analysis with association between green tea consuming with chemoprotection to tumor [89]. Epigallocatechin-3-gallate (EGCG) (**20**) represents the main active ingredient in green tea [90,91]. Several antitumor mechanisms were reported, including apoptosis activation, initiation of cellular growth block, modification of cellular life regulatory proteins, induction of killer caspases, and NF-kB pathway inhibition [89,90]. EGCG attenuated molecular pathways implicated in tumor advance [92] and in modulation of the immune system in murine cancers [93,94,95,96]. Rawangkan et al. [97] hypothesized that EGCG inhibited PD-L1, a checkpoint molecule, increasing anticancer immune response [97].

Resveratrol (**21**, Figure 3), a non-flavonoid polyphenolic substance present in grapes and red wine, modulates lipoprotein metabolism [98,99], eicosanoid production [100,101,102], lipid oxidation [103], platelet aggregation [100], and inhibits cyclooxygenase-2 (COX-2) [104]. Recently, it was also demonstrated that **21** arrested cell processes related to cancer induction, in vivo expansion and in vitro development of pre-neoplastic lesions in mice mammary glands [105]. In several studies, resveratrol has demonstrated to impede expansion of breast, oral, liver, prostate, and colon tumor cell lines [106,107,108,109]. This activity has been linked to the ability of resveratrol to block citokyne production (IFN-γ, IL-2, TNF-α and IL-12) and the activation of transcription factor NF-kB [110]. This compound also suppressed IL-6, IL-10, and interleukin 1 (IL-1) secretion in human PBMCs with tumor promoter activity [111]. Several clinical trials to investigate potential of resveratrol in cancer disease are available (Resveratrol Clinical Trials. Available online: https://clinicaltrials.gov/ct2/results?cond=Cancer&term=resveratrol&cntry=&state=&city=&dist= (access on 13 April 2022)).

### 2.2. Therapeutic Antitumor Activity and Natural Compounds from Spices

**Figure 4:**
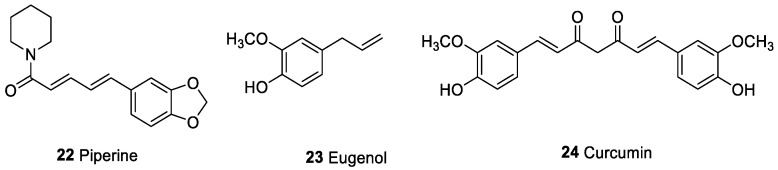
Potential active compounds from spices (black pepper, cardamom, and curcuma).

Natural spices have been the subject of many recent studies on the discovery of antitumor agents. In 2009, Majdalawieh and Carr investigated in vitro immunomodulatory and anticancer activities of black pepper (*Piper nigrum*) and cardamom (*Elettaria cardamomum*) [112]. These spices possess great potential in the prevention and treatment of different pathologies, including tumors [24,113,114]. The authors suggested that the anticancer activity of black pepper and cardamom extracts could be due to activation of NK cells [112]. However, the immunomodulatory influence of spices on the secretion of main cytokines by splenocytes and macrophages were not deeply analyzed. In this in vitro analysis, the production of T helper 2 cytokines (Th2), interleukin 4 (IL-4), and IL-10 was hindered by black pepper extracts and favored by cardamom extracts. On the contrary, the secretion of Th1 cytokine IFNγ was increased and decreased by black pepper and cardamom aqueous extracts, respectively, suggesting that these extracts contained compounds capable to promote proliferative signaling pathways in splenocytes. Piperine (**22**, Figure 4), an alkaloid of black pepper, enhanced murine splenocyte proliferation [115], induced anti-proliferative effects on human colon tumor cells [116], and in vivo antitumor activity [117,118,119,120,121]. Piperine alone did not elicit any cytotoxicity; 50% loss in cell was observed when the alkaloid at 1 μg/mL was added to cadmium [115]. Similarly, eugenol (**23**, Figure 4), also contained in cardamom, enhanced in vitro lymphocyte expansion [122] and inhibited in vivo tumor formation [123,124,125,126].

Curcumin, a polyphenol isolated from the plant *Curcuma longa*, called turmeric (**24**, Figure 4), is a substance widely analyzed for the immunomodulatory and antitumor activities [127,128]. Various preclinical investigations showed antitumor activity of curcumin by modulation of tumor mediators, such as NF-kB, COX-2, LOX, and PKC [129,130]. Curcumin inhibited the accumulation of myeloid-derived suppressor cells (MDSC) and their differentiation and interaction with tumor cells, and induced MDSC differentiation [126,128]. These properties are potential strategies for cancer prevention and therapy. Furthermore, curcumin decreased intra-tumor IL-6 secretion and metastasis production in a breast tumor model [131]. In RAW 264.7 murine macrophages, curcumin reduced the lipopolysaccharides (LPS)-induced inflammatory signals NO, PGE2, and IL-6 by NF-kB deactivation [132]. Moreover, curcumin inhibited the expression and functionality of indoleamine-2,3 dioxygenase (IDO), a main immunosuppressive enzyme in cancer immunology and reduced inflammation-mediated PDL-1 expression [133,134]. Several clinical studies are available on the application of curcumin in oncological diseases (alone or in combination with chemotherapeutic drugs) (Curcumin Clinical Trials. Available online: https://clinicaltrials.gov/ct2/results?cond=Cancer&term=curcumin&cntry=&state=&city=&dist= (accessed on 13 April 2022)). 

A main hurdle for the development of curcumin in cancer treatment is its scarce natural availability [135]. To study the role of oxidative activation in protein adduction of curcumin, a stable and oxidizable alkynyl-tagged analog was prepared, the 3′-*O*-Alkynyl-5′-*O*-methoxycurcumin, very similar to curcumin in the autoxidation potency (1.6 ± 0.4 µM/min v.s. 4.0 ± 0.4 µM/min) and IC50 for NF-ĸB inhibition (24 v.s. 18 µM) [127].

Although black pepper, cardamom, and curcumin are promising as immunomodulatory and chemopreventive molecules, their molecular mechanisms are still under investigation.

### 2.3. Macromycetes

**Figure 5:**
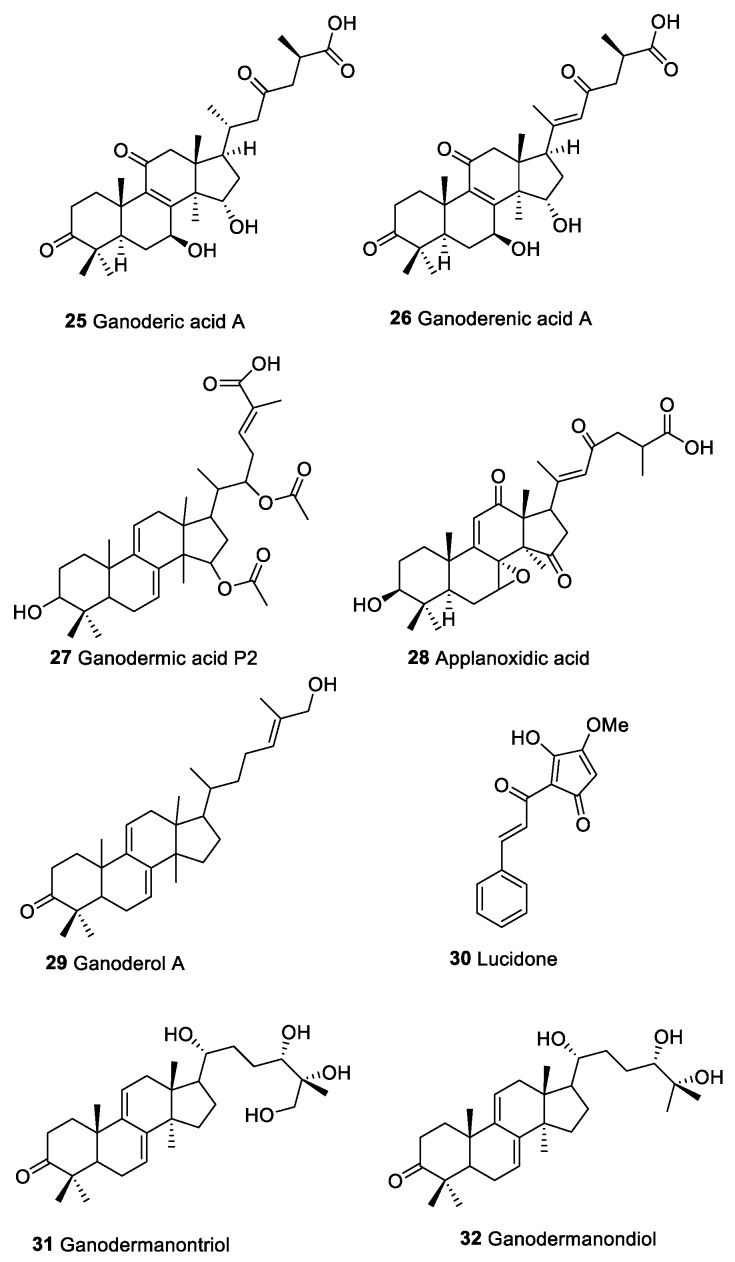
Triterpenoid compounds from macromycetes with anticancer and immunomodulatory properties.

Macrofungi are renowned source of macro- and small molecules characterized by different immunomodulatory and antitumor activities, including polysaccharides, glycopeptide, proteoglycans, proteins, and terpenoids [136]. In particular, β-(1→3)-d-glucans along with their peptide analogs and fungal immunomodulatory proteins (FIPs) play an important role in immunomodulatory and anticancer activity. The bioactivity of these molecules is linked to their potential impact toward immune effector cells as hematopoietic stem cells, lymphocytes, macrophages, T cells, DCs, and NK cells involved in the innate and adaptive immunity [136,137]. Even if a considerable quantity of investigations on biological mechanisms of macrofungi macromolecules were published, few works investigated on the anticancer and immunomodulatory effects of terpenoids isolated from this source. Triterpenoids (Figure 5) such as ganoderic acids (**25**), ganoderenic acids (**26**), ganodermic acids (**27**), applanoxidic acids (**28**), ganoderols (**29**) lucidone (**30**), ganodermanontriol (**31**), and ganodermanondiol (**32**) have been isolated exclusively from macrofungi and higher fungi (Basidiomycetes) [138]. These molecules show highly oxidized lanostane structures with anti-infectives, cytotoxic, and immunomodulatory activities [100]. Studies on *Ganoderma lucidum* underlined that the anticancer property of some terpenoids was comparable to that of β-d-glucans in the capability to induce NF-kB pathway and regulate Ras/Erk, c-Myc, cAMP response element-binding protein (CREB) protein and MAPK [139].

### 2.4. Other Sources

The honeybees secrete the propolis, long used in traditional medicine. Propolis show antiviral, antibacterial, antifungal, anti-inflammatory, hepatoprotective properties, and cytotoxic activity against malignant tumor cell lines [140,141]. This substance also posseses immunomodulatory activity [141,142] and indirect antitumor activity by increasing the host’s defense against neoplastic growth via macrophage activation [143]. Discrepancy in bioactivity of propolis of diverse geographic areas suggested differences of the chemical constituents due to plant variety [144]. It has been reported that propolis from Australia attenuated skin inflammation through immunosuppressant property and reduction of lipid peroxidation in the setting of chronic skin inflammation [145]. It can also suppress the classic photo-immune reaction by cutting down the inflammatory cytokines IL-6 and IL-12 and by overexpression of the anti-inflammatory IL-10 [145].

**Figure 6:**
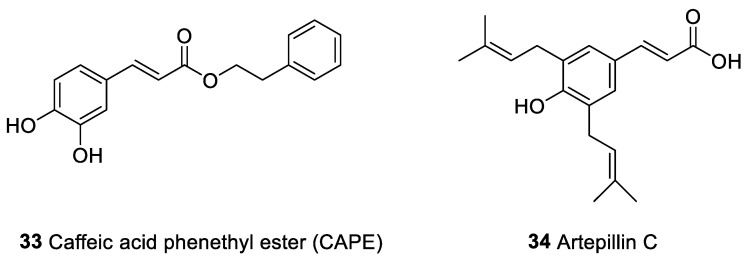
Potential anticancer and immunomodulatory compounds from propolis.

Caffeic acid phenethyl ester (CAPE) (**33**, Figure 6) is one of chemical compounds identified from propolis [146] with anti-inflammatory and antitumor properties [147,148,149]. The anticancer activity of CAPE is due to the impairment of DNA synthesis [150], block of transduction signaling growth [151], induction of apoptosis through intrinsic apoptotic pathway and priming of antiangiogenic activity [152,153]. CAPE strongly inhibits T cell expansion [154] and reduces the binding and DNA transcriptional functions of NF-kB and nuclear factor of activated T-cells (NFAT), key transcriptional factors for T cell activation. CAPE inhibits DCs maturation after LPS engagement [155,156]. It has been shown that this compound was able to inhibit the release of IL-2 in a concentration-dependent manner (with an IC_50_ of approximately 1 µM) [154]. CAPE was cytotoxic to cancer or virally transformed cells [155,157,158] but not to normal cells. Lee et al. [159] found that the molecule exhibited strong antitumor effects in oral cancer cells [159].

A further interesting compound present in propolis is artepillin C (hydroxycinnamic acid derivative) (**34**, Figure 5), which is reported to kill tumor cells indirectly by amplifying T cell-mediated cytotoxicity and inhibiting NF-kB activity in macrophages [160]. The influence of artepillin C on the reactivity of inflammatory cells was demostrated in vitro model on the macrophage cell line RAW 264.7 and an IC_50_ of 8.5 (7.8–9.2) μM was estimated. Nevertheless, immunogenic properties of artepillin C were less studied than that of CAPE [160].

**Table 1:** Terrestrial occurring immunomodulators with antitumor activity.

Molecule	Type of Compound	Source	Tumor	Immuno System’s Role	References
Andrographolide (**1**)	Terpene	*Andrographis paniculata*	Human epidermal carcinoma (KB, ED_50_ 1.5 µg/mL); lymphocytic leukemia (P388, ED_50_ 1.0 µg/mL)	Stimulate antigen specific and non-specific immune responses in mice	[29]
Triptolide (**5**)	Terpene	*Chinese Tripterygium Hook F (TWHF)*	Solid tumor cells	Apoptosis induced by TNFα, inhibition of NF-kB	[44]
Zerumbone (**4**)	Terpene	*Zingiberaceae*	Human cancer cell lines of the ovary (Coav-3) breast (MCF-7) promyelocytic leukemia (HL-60) and colon adenocarcinoma HCT116	Immunosuppressive effects via inhibition of AP-1 and NF-kB	[36,37,38,39]
β-Carotene (**6**); Lutein (**7**)	Carotenoids	Plant	Lung human cancer, mammary tumor bearing mice model	Stimulate NK cell activities, increase the number of leukocyte immune cells, CD4/CD8 ratio, and surface expression of MHC I molecules Stimulation effect on IFN-γ mRNA expression; suppression of IL-10 in splenocytes	[47,48,49]
β-Sitosterol (**8**)	Terpene	Plant	Human cancer cell line of the colon (HT-29) and prostate (LNCaP)	stimulated blood lymphocyte proliferation in vitro; enhanced lytic and cytotoxic activities of NK cells	[53,161]
Wogonin (**9**), Baicalein (**10**), Baicalin (**11**)	Flavones	*Scutellaria baicalenis*	Breast, prostate, and lung human cancer	Activation of NF-kB factor; cell cycle regulation	[60]
Geraniin (**12**),	Phenolic compounds	*Phyllanthus emblica Linnaeus*	Human cancer cell line of the breast (MCF-7) and embryonic fibroblast (HELF)	Promoted the level of serum IL-18 and NK cell cytotoxicity, suggesting stimulation of macrophages, thereby upregulating the NK cell-mediated antitumor immune response	[79]
Kaempferol 3-β−d-glucopyranoside (**13**)					
Kaempferol (**14**)					
Quercetin 3-β-d-glucopyranoside (**15**)					
Quercetin (**16**)					
Isocorilagin (**17**)					
Ruitin (**18**)	Phenolic compounds	*Diospyros kaki* L.	Leukemia, colorectal, neuroblastoma, melanotic melanoma and prostate human cancer.	Increase of IL-18; Upregulation of NK cells	[83,84,85]
Myricetin (**19**)					
Epigallocatechin-3-gallate (**20**)	Phenolic compound	*Camelia sinesis*	Human lung cancer cell lines	Induction of apoptosis and suppression of NF-kB pathway Inhibition of PD-L1	[89,90,91,97]
Resveratrol (**21**)	Phennolic compound	Grapes and red wine	Brest, oral, liver, prostate and colon human cancer	Inhibition of citokyne production, (IFN-γ, IL-2, TNF-α and IL-12); block the activation of transcription factor NF-kB	[104,105,106,110]
Piperine (**22**)	Alkaloid	*Piper nigrum*	Human colon cancer cell lines	Cytotoxic activity of NK cells; suppression of the relase of Th2 cytokines IL-4 and IL-10; enhance murine splenocyte proliferation	[112,115,116,117,121]
Eugenol (**23**)	Phenolic compound	*Cardamom*	Inhibition of tumor formation in vivo	Cytotoxic activity of NK cells; suppression of the relase of Th2 cytokines IL-4 and IL-10	[112,123,124,125,126]
Curcumin (**24**)	Phenolic compound	*Curcuma longa*	Human breast cancer	Modulation of NF-kB; reduction of IL-6; inhibit inflammation-mediated PD-L1 expression	[129,130,131,133,134]
Ganoderic acids (**25**)	Triterpenoid compounds	Macromycetes		Act on immune effecter cells such as hematpoietic stem cells, lymphocytes, macrophages, T cells, DCs, and NK cells Activation NF-kB pathway and modulate Ras/Erk, c-myc, CREB protein and MAPK	[139]
Ganoderenic acids (**26**)					
Ganodermic acids P2 (**27**)					
Applanoxidic acid (**28**)					
Ganoderol A (**29**)					
Lucidone (**30**)					
Ganodermanontriol (**31**)					
Ganodermanondiol (**32**)					
Caffeic acid phenethyl ester (CAPE, **33**)	Phenolic compounds	Propolis	Oral human cancer and human cancer cell lines of the promyelocytic leukemia (HL-60)	Inhibition of T cell receptor-mediated T cell proliferation	[150,151,154,158,159]
Artepilin C (**34**)					

Abbreviations: AP-1, activator protein 1; NF-kB, nuclear factor kappa of activated B cells; CD, cluster of differentiation; MHC, Major histocompatibility complex; IFN-γ, interferon-gamma; IL-2, interleukin 2; IL-4, interleukin 4; IL-6, interleukin 6; IL-10, interleukin 10; IL-12, interleukin 12; IL-18, interleukin 18; NK, natural killer; TNFα, tumor necrosis factor α; PD-L1, Programmed death-ligand 1; Th2, T helper 2; DC, dendridic cell; CREB, cAMP response element-binding protein; MAPK, mitogen-activated protein kinase; ED_50_, Median Effective Dose.

## 3. Compounds from Marine Environment

The sea, covering a large part of Earth surface, is a basin of huge biological diversity, with more than three hundred thousand species of living organisms described until now [162,163]. The hostile and competitive marine habitat makes these organisms a great depot of molecules with biological properties undiscosed in terrestrial environments. In Table 2, were summarized MNPs with immunomodulatory and antitumor properties herein cited.

Marine macroalgae (especially brown algae) contain a relevant amount of soluble biologically active polysaccharides, such as alginates and fucoidans, and small molecules (tripeptides, phlorotannins, glycolipids, and carotenoids) that have a potential function as dietary fiber and are also used as immune activators [164]. Mekabu is an edible alga exerting anticancer and immunogenic effects [164]. In vivo studies of activity of fucoidans found in Mekabu highlighted the reduction of the tumor growth through Th1 and NK cell responses [165]. Carrageenans, a family of sulfated galactans isolated from marine red algae, exhibit significant antitumor and immunomodulatory activities at different extent [164]. Several studies showed that the molecular weight (MW) of these polysaccharides affect their activity [166,167]. The highest inhibition rate on sarcoma S180 and hepatoma H22 were recorded with MW between 9.3 and 15 kDa, with 66.15 and 68.97% inhibition at the dose of 200 mg kg^−1^ per day, respectively. The antitumor activities of these compounds are not due to cytotoxic effects, thus the authors put forward the activation of body immunocompetence to explain the results.

From the marine dinoflagellate *Alexandrium minutum* has been identified a glycopeptide (Table 2) that induces mitophagic cell death in the cancer cell line without affecting normal cell line viability [162]. This mechanism causes the lysosomal secretion of ATP, which stimulates myeloid cells and can induce immunogenic cell death [21].

Similarly, the polyunsaturated short-chain aldehydes produced by *Thalassiosira rotula*, *Skeletonema costatum*, and *Pseudonitzschia delicatissima* [167,168,169,170,171] (Table 2), cause specific programmed cell death in lung and colon adenocarcinoma, inducing the release of ATP and other immune signals, which are known as ICD inducers [21].

**Figure 7:**
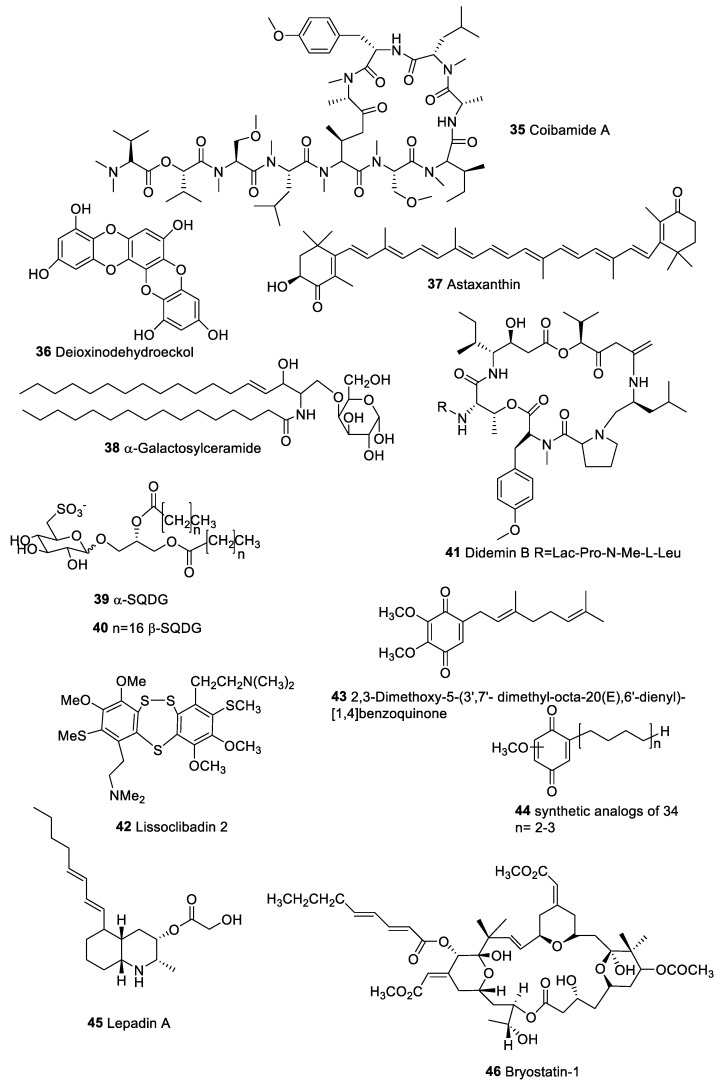
Anticancer and immunomodulatory compounds from marine environment.

The depsipeptide coibamide A (**35**, Figure 7), isolated from the marine cyanobacterium *Leptolyngbya* sp. induces caspase-independent cell death in breast cancer cells with EC_50_ at nanomolar level [168]. As a consequence of severe lysosome defects, the autophagosome-lysosome fusion was blocked upon treatment with coibamide A. Autophagy is responsible for the release of DAMPS [21].

Dioxinodehydroeckol (**36**, Figure 7), phlorotannin isolated from seaweed *Ecklonia cava*, exhibited a remarkable antiproliferative effect on human breast cancer cells (MCF-7). This activity has been associated with the induction of apoptosis through activation of the transcription factors of the NF-kB family [169].

Astaxanthin (**37**, Figure 7) is a carotenoids with chemopreventive activity found abundantly in seaweeds [170]. The molecule improves antitumor immune responses by inhibiting lipid peroxidation induced by stress [170]. Astaxanthin protected mice from carcinogenesis of the urinary bladder by reducing the incidence of chemically induced bladder carcinoma [171]. Dietary astaxanthin also exerted antitumoral activity in the post-initiation phase of carcinogen-induced colon [172] and oral cancer models [173].

Sponges represent a primary source of bioactive marine substances. Galactosylceramide (α-GalCer) (**38**) was the first active marine glycolipid derived from sponges (not occurring in mammalian cells). This lipid stimulates NKT cells to produce both Th1 and Th2 cytokines and shows antitumor effects in mice [174,175]. The antitumor properties are mediated by CD1d-restricted iNKT cells that activate NK and antigen presenting cells (APCs) to stimulate antitumor immune responses [174,175]. Clinical trials to evaluate the therapeutical application have been already completed (Galactosylceramide Clinal Trials. Available online: https://clinicaltrials.gov/ct2/results?cond=&term=Galactosylceramide+&cntry=&state=&city=&dist= (accessed on 13 April 2022)).

Screening of marine extracts on monocyte-derived human dendritic cells (hMo-DCs) provided positive results for α-sulfoquinovosildiacylglycerols (SQDGs **39**, Figure 7), plastidial sulfolipids occurring in algae and other photosynthetic organisms [176]. Natural SQDGs showed moderate activity but a rational change of their chemical structure led to the synthetic analogues β-sulfoquinovosildiacylglycerols (SULF A **40, Figure 7**) wich induces DCs maturation and triggers in vivo immune resposte. The product was tested as adjuvant in an experimental model of tumor vaccine against a murine B16F10 melanoma cell line with very encouraging results [176]. Recent studies on the mechanism of action indicated that SULF A is a ligand of the Triggering Receptor Expressed on Myeloid cells-2 (TREM2) [177]. The TREM2-iduced response is mediated by SYK-NFAT axis and is compromised by blockade and gene silencing of the receptor. Activation by this lipid preserved the DC functions to excite the allogeneic T cell response and promoted interleukin-10 (IL-10) release after lipopolysaccharide (LPS) stimulation [177].

A few successful antitumor compounds derived from tunicates and ascidians have made significant progress through clinical trials in the USA and Europe. Didemnins are bioactive depsipeptides, first isolated from the Caribbean tunicate *Trididemnum solidum*, exhibited various biological properties [178]. Didemnin B (**41**, Figure 7) shows cytotoxic activity against L1210 murine leukemia cells at very low concentrations, as well as has an immunosuppressant activity through inhibition of the lymphocyte activation at concentrations of 10 pg/mL (IC_50_) [179,180]. In 2011, Tsukimoto et al. reported the identification of the molecule from the marine α-proteobacteria *Tistrella mobilis* and *Tistrella bauzanensis* [181] that uses a unique post-assembly line maturation process [182]. Didemnin B was the first marine-derived product to advance into clinical trials (phase I and phase II) in the early 1980s. Due to several side effects for an unpredicted toxicity and short half-life [179], these trials were officially terminated in the middle 1990s. Recently anticancer marine peptides have been reviewed by Zhang et al. [183]. Cyclic peptides have significant structural advantages, displaying a large surface area, which provides a high affinity and selectivity for protein targets. Thus, they are simple to modify, handle, and characterize, all essential properties for therapeutics [183]. Aplidin^®^ (dehydrodidemnin B), a second generation didemnin isolated from the Mediterranean tunicate *Aplidium albicans*, was more effective and less toxic than Didemnin B in clinical trials [184]. The molecule was granted “orphan drug status” in the European Union for acute myeloblastic leukemia. Recent studies on the treatment of adults with SARS-COV-2 requiring hospitalization suggest that, in addition to its antiviral effect [185], dehydrodidemnin B could stimulate the immune response against the virus.

Lissoclibadin 2 (**42**, Figure 7), a trimeric compound isolated from the tunicate *Lissoclinum cf. badium* ahowed inhibitory activity, efficient against human colon tumor cells DLD-1 and HCT116, breast tumor cells MDA-MB-231, renal tumor cells ACHN, and non-small-cell lung tumor cells NCI-H460. Moreover, the molecule increases IL-8 production, indicating a link between cancer-killing and the immune system [186,187]. The benzoquinone derivative (**43**, Figure 7), found in *Aplidium glabrum* and its synthetic analogues (**44**, Figure 7), induced apoptosis in JB6 CI41 tumor cell lines and inhibited p53 while increasing AP-1 and NF-kB transcription. Inhibition of cellular modification was shown to strictly depend on terpenoid side chain length [186,188].

Lately, we have identified by bioassay-guided fractionation the alkaloid lepadin A (**45**) from the tunicate *Clavelina lepadiformis* sp. B, already known to exhibit significant in vitro cytotoxicity against several human cancer cell lines, as a potent activator of innate immune cells [189]. In this study, lepadin A showed both cytotoxic effect against chronic forms of lung carcinoma, melanoma, and multiple myeloma cells together with maturation of mouse dendritic cells at micromolar concentrations. The combination of the two effects is expected to increase the anticancer properties by a synergistic mechanism deriving from both the role of mature DCs in the generation of antitumor activity and the release of immunogenic molecules by dying cells. In particular, the marine alkaloid triggers a significant over-expression of MHC-II and co-stimulatory molecules that are key signals for naïve T cell differentiation by DCs and for mounting an effective immune response. Moreover, the immune response of **45** occurs at subtoxic concentrations (EC_50_ = 1.64 µg/mL; IC_50_ = 4.20 µg/mL), indicating that the molecule can induce a cell stress. On this basis, even though further studies are needed to investigate the mechanism of action, it was suggested that lepadin A could act as ICD inducer [189].

Although their constituents and peculiar activities are still to be studied, the extracts from marine organisms are often tested for immunostimulatory activity together withtumor prophylaxis and therapy. For example, in vitro and in vivo antitumor and immunomodulatory activities of the ethanolic extract of the simple ascidian *Microcosmus exasperates* [190] and *Phallusia nigra* [191] have been reported. Sea cucumbers metabolites have been also proposed as potent anticancer agents and raw extracts of these marine organisms can suppress inflammation and increased innate immune responses [192]. Frondanols (sulfated triterpenoid glycoside), frondosides (sulfated glycoside), eicosapentaenoic acid, 12-methyltetradecanoic acid, and fucosylated chondroitin sulfate, as well as canthaxanthin/astaxanthin (detected in little amount in the extracts), are the main bioactive metabolites of sea cucumbers with immunomodulatory properties, ameliorating immune reaction by modulation of innate immune cells [192].

Bryostatin-1 (**46**, Figure 7), a macrocyclic lactone of the marine Bryozoan, *Bugula neritina* [193], show antitumor and immunomodulatory activity and it is currently in clinical trial [194]. The molecule is a potent activator of PKC class, lacks cancer-inducing efficacy, and shows antagonistic activity on cancer-inducing phorbol esters. This activity is presumably linked to PKC down-regulation or to peculiar isoform activation. The molecule also induced cytokines secretion, bone marrow progenitor cells, and neutrophils [195,196,197]. Increase in IL-2-promoted proliferative response in PBLs was detected, but not upregulation of IL-6 or tumor necrosis factor (TNF). In vitro, **46** displayed cytotoxic effects toward several leukemia and solid tumor lines [196]. It has also in vivo antitumor activity in different mouse models, as leukemia, lymphoma, ovarian cancer, and melanoma. The potential of Bryostatin-1 on several tumors has been reported by many clinical studies (Bryostatin-1 Clinical Trials. Available online: https://clinicaltrials.gov/ct2/results?cond=cancer&term=Bryostatin-1+&cntry=&state=&city=&dist= (accessed on 13 April 2022)).

Trabectidin, a tetrahydroisoquinoline alkaloid that was initially isolated from the Caribbean tunicate *Ecteinascida turbinata* with the name of ET-743 [198,199], has been the first marine-derived anti-neoplastic drug approved for the treatment of advanced soft tissue sarcoma and, in combination with pegylated liposomal doxorubicin, for the treatment of patients with relapsed platinum-sensitive ovarian cancer. The alkaloid besides the cytotoxic effect has immunomodulatory activity on several cell types of the microenvironment [200]. Several studies have also underlined that Trabectedin is an immunomodulatory drug with potential use in enhancing the therapeutic response to checkpoint inhibitor-based immunotherapy and in overcoming chemoimmune resistance [200,201]

**Table 2:** Marine occurring immunomodulators with antitumor activity.

Molecule	Source	Tumor	Immuno System’s Role	References
Glycopeptide	*Alexandrium minutum*	A549 Lung adenocarcinoma cell line	Mitophagy and ICD inducer	[202]
Polyunsaturated aldehydes	diatoms	Programmed cell death in lung and colon adenocarcinoma	Induce the release of ATP and others immune signals which are known as ICD inducers	[21,163,202,203,204,205]
Coibamida A (**35**)	*Leptolyngbya* sp.	Breast camcer	Caspase-independent cell death and ICD inducer	[168]
Dioxinodehydroeckol (**36**)	*Ecklonia cava*	Human cancer cell line of the breast (MCF-7)	Induction of apoptosis through NF-kB family and NF-kB-dependent pathway	[169]
Astaxanthin (**37**)	Seaweeds	Antitumoral activity in the post-initiation phase of carcinogen-induced colon and oral cancer models	Improves antitumor immune responses by inhibiting lipid peroxidation induced by stress	[170,171,172,173]
α Galactosylceramide (**38**)	Sponge	Antitumor effects in mice	Stimulation of NKT cells to produce both Th1 and Th2 cytokines	[174]
α-Sulfoquinovosides (**39**)	Marine microalgae	Synthetic β-sulfoquinovosides derivative as adjuvant in vaccine against a murine B16F10 melanoma cell line	Maturation of human DCs.	[176,177]
Didemin B (**41**)	*Trididemnum solidum*		Inhibition of lymphocyte activation	[181,182,183]
Lissoclibadin 2 (**42**)	*Lissoclinum cf. badium*	Human colon cancer lines (DLD-1) and (HCT116), breast cancer lines MDA-MB-231, renal cancer line ACHN; non-small-cell lung cancer line NCI-H460	Increase of IL-8 production	[187]
2,3-Dimethoxy-5-(3′,7′-dimethyl-octa-20(E),6′-dienyl)-[1,4] benzoquinone (**43**)	*Aplidium glabrum*	JB6 CI41 cancer cell	Inhibition of p53; Increase transcription of AP-1 and NF-kB	[186,188]
Lepadin A (**45**)	*Clavelina lepadiformis* sp. B	Human lung carcinoma, melanoma, and multiple myeloma	Mouse DCs	[189]
Bryostatin 1 (**46**)	*Bugula neritina*	Antitumor activity against leukemia, lymphoma ovarian cancer, and melanoma	Activation of PKC family; Stimulation of cytokine production	[193,194]

Abbreviations: AP-1, activator protein 1; NF-kB, nuclear factor kappa of activated B cells; CD, cluster of differentiation; IL-8, interleukin 8; NK, natural killer; Th1, T helper 1; Th2, T helper 2; DC, dendridic cell; PCK, Protein kinase C.

## 4. Conclusions: Future Prospects of Natural Compounds as Potential Anti-Cancer Agents

Cancer is one of the most challenging medical conditions and requires continuous updating of therapeutic approach. Many natural products have been tested for their anticancer potential and some of these compounds have successfully advanced in clinical trials. Even today, natural molecules continue to offer novel chemicals with a wide range of mechanisms of action to help the world fight cancer.

However, due to the serious collateral effects of the traditional cancer chemotherapy, immunotherapeutic treatments are becoming increasingly widespread. The goal of immunotherapy is to fight cancer by stimulating the immune system, our body’s natural defense system. Immune system cells usually activate themselves against anything they recognize as non self, for example pathogens or mutated cells in order to eliminate them. Regrettably, tumor cells use a number of mechanisms to evade this control, increase in number and spread out in the body. Cancer immunotherapy has demonstrated to prevent these self-masking mechanisms of tumor cells, so that the immune system is no longer cheated. The key breakthrough in the development of cancer immunotherapy has been the discovery of immune checkpoints as molecules involved in the tumor immune evasion. Very important therapeutic results have already been achieved with checkpoint inhibitors in melanoma, non-small-cell lung carcinoma, and urothelial cancer [20].

Immunomodulatory compounds and antitumor molecules of natural origin have attracted great interest for the potential to promote the activation and recruitment of immune cells as macrophage, neutrophils, dendritic cells, NKs, and T lymphocytes lead to infiltration in the cancer microenvironment with effector cells facilitating the cancer eradication. In addition, natural products with antioxidant and anti-inflammatory activity, are used to potentiate the immune response and to prevent induction and formation of tumors.

Most of these studies are related to natural compounds from terrestrial sources. However, micro and macro marine organisms are receiving increasing attention in this context and represent a promising platform for the development of new candidates for anticancer immunotherapy.

## Abbreviations

AP-1: activator protein 1; APCs, antigen presenting-cell; CASP3, caspase-3; CTX, Cyclophosphamide; COX, cyclooxygenase; COX-2, cyclooxygenase-2; CREB, cAMP response element-binding protein; CTL, cytotoxic T lymphocyte; DC, dendridic cell; Fips, fungal immunomodulatory proteins; hMO-DCs, monocyte-derived human dendritic cells; IAPs, inhibitor of apoptosis proteins; IFN-γ, interferon γ; IL-1, interleukin 1; IL-2, interleukin 2; IL-4, interleukin 4; IL-6, interleukin 6; IL-8, interleukin 8; IL-10, interleukin 10; IL-12, interleukin 12; IL-18, interleukin 18; iNOS, inducible nitric oxide synthase; IDO, indoleamine-2,3 dioxygenase; JAK2, janus kinase 2; LOX, lipooxigenase; LPS, lipopolysaccharides; MAPK, mitogen-activated protein kinase; MAPK/PKB, mitogen-activated protein kinase/protein kinase B; MDSC, myeloid-derived suppressor cells; CNP, natural product; ND, natural derivate; NK, natural killer; NF-kB, nuclear factor kappa of activated B cells; NO, nitric oxide; NFAT, nuclear factor of activated T-cells; NSCLC, non-small-cell lung cancer; PGE2, prostaglandin E2; PLF, flavonoids extract from persimmon leaves; PK, protein kinase; PKB, protein kinase B; STAT3, signal transducer and activator of transcription 3; TME, tumor immune microenvironment; TPA, tissue polypeptide antigen; TNF, tumor necrosis factor; TNFα, tumor necrosis factor α; TNFc, tumor necrosis factor c; TRAIL, TNF-related apoptosis-inducing ligand; TH, T helper; TH1, T helper 1; TH2, T helper 2; WHO, world health organization.
